# OHDSI-compliance: a set of document templates facilitating the implementation and operation of a software stack for real-world evidence generation

**DOI:** 10.3389/fmed.2024.1378866

**Published:** 2024-05-16

**Authors:** Felix N. Wirth, Hammam Abu Attieh, Fabian Prasser

**Affiliations:** Berlin Institute of Health at Charité – Universitätsmedizin Berlin, Center of Health Data Science, Berlin, Germany

**Keywords:** health data analytics, real-world evidence, observational health data science, regulatory compliance, data protection

## Abstract

**Introduction:**

The open-source software offered by the Observational Health Data Science and Informatics (OHDSI) collective, including the OMOP-CDM, serves as a major backbone for many real-world evidence networks and distributed health data analytics platforms. While container technology has significantly simplified deployments from a technical perspective, regulatory compliance can remain a major hurdle for the setup and operation of such platforms. In this paper, we present OHDSI-Compliance, a comprehensive set of document templates designed to streamline the data protection and information security-related documentation and coordination efforts required to establish OHDSI installations.

**Methods:**

To decide on a set of relevant document templates, we first analyzed the legal requirements and associated guidelines with a focus on the General Data Protection Regulation (GDPR). Moreover, we analyzed the software architecture of a typical OHDSI stack and related its components to the different general types of concepts and documentation identified. Then, we created those documents for a prototypical OHDSI installation, based on the so-called Broadsea package, following relevant guidelines from Germany. Finally, we generalized the documents by introducing placeholders and options at places where individual institution-specific content will be needed.

**Results:**

We present four documents: (1) a record of processing activities, (2) an information security concept, (3) an authorization concept, as well as (4) an operational concept covering the technical details of maintaining the stack. The documents are publicly available under a permissive license.

**Discussion:**

To the best of our knowledge, there are no other publicly available sets of documents designed to simplify the compliance process for OHDSI deployments. While our documents provide a comprehensive starting point, local specifics need to be added, and, due to the heterogeneity of legal requirements in different countries, further adoptions might be necessary.

## 1 Introduction

### 1.1 Background

Collecting and analyzing data from real-world healthcare settings at a broad scale can provide new insights into patient outcomes, treatment efficacy, and healthcare practices (1). This usually necessitates bringing together data from several healthcare institutions, which requires the implementation of or mapping to data standards, as well as approaches for ethical and data protection compliant access (2). One common solution for the latter challenge is federation, where the analysis is brought to the data instead of bringing the data to the analysis (3). This is, for example, implemented by SHRINE (4), DataSHIELD (5) and the Observational Health Data Sciences and Informatics (OHDSI) (6) initiative. OHDSI is an international, multidisciplinary community of researchers and healthcare professionals to enable data standardization, analysis, and insight discovery from large-scale health datasets, launched in 2013. The community distributes a set of open-source software tools to represent and analyze data in the Observational Medical Outcomes Partnership (OMOP) Common Data Model (CDM), which makes extensive use of terminologies and ontologies, such as Logical Observation Identifiers Names and Codes (LOINC) or Systematized Nomenclature of Medicine (SNOMED) Clinical Terms (CT) (7). While the term OMOP describes the now discontinued collaboration that originally developed the CDM, the term OMOP-CDM refers to the further developed version that forms the current technical cornerstone of OHDSI. The EHDEN project has funded the deployment of the OMOP-CDM and the OHDSI software stack across Europe (8). Moreover, the OMOP-CDM will also play an important role in the upcoming European Health Data Space (EHDS; see Section “Discussion”). The EHDS is planned as a large-scale ecosystem facilitating better exchange and access to different types of health data throughout the European Union (EU). EHDS pillar I focuses on primary healthcare data use, i.e., data sharing for healthcare delivery. EHDS pillar II focuses on secondary use of health data, e.g., analysis for research, policy-making or drug safety (9).

Setting up an OHDSI node can involve significant efforts, in particular for the required mapping to standards. However, technical and data integration challenges are not the only obstacles faced when connecting to data sharing networks [for one example for the various technical challenges see (10)]. Legal and regulatory compliance is another important issue (11, 12). National and international data protection laws as well as ethical guidelines must be considered. Important examples include the US Health Insurance Portability and Accountability Act (HIPAA) (13) and the European Union (EU) General Data Protection Regulation (GDPR) (14). To fulfill central requirements, concepts need to be developed and documented for ensuring the confidentiality of the processed healthcare data. An important example is the so-called Record of Processing Activities (ROPA), which needs to be created according to the GDPR, but also according to laws in the United Kingdom (15, 16), Australia (17) or Thailand (18). Amongst other aspects, a ROPA typically describes the processed categories of data and details information flows as well as the technical and organizational security measures implemented, although slight variations might exist between the requirements in different countries. Moreover, information security plays an important role, with relevant standards also requiring documentation of the measures taken (19). Important examples include the International Standards Organization (ISO) Standard 27001 (20), (2) the US National Institute of Standards and Technology (NIST) Cybersecurity Framework (21) or (3) the Health Information Trust Alliance Common Security Framework (HITRUST CSF) (22).

### 1.2 Objective

It is well known that conceptualizing and documenting the secure operation of data processing platforms can be challenging (23, 24). Research has shown that even reading and comprehending such documents can be difficult (25–27). As a result, different guidelines and templates have been developed (see Section *Comparison with prior work*). However, those are usually generic in nature and not directly applicable to the establishment of an OHDSI node. The objective of the work described in this paper, was to conceptualize an approach specifically for common OHDSI deployments. Moreover, we developed document templates that can be customized to local requirements. We focus on documents for a general OHDSI setup. Depending on the nature of projects that use this infrastructure as well as local requirements, additional documents might be needed for the individual studies performed.

## 2 Methods

### 2.1 Overview of the OHDSI tools

The main tools provided by OHDSI are focused on (1) establishing a common data model with clearly defined structure and semantics, as well as (2) assisting medical researchers and data scientists in extracting knowledge from this data. The OMOP-CDM is the central pillar of OHDSI, providing a standardized database schema and a set of terminologies with which heterogeneous data from different sources can be integrated to provide comparability across studies and institutions (28). As a result, OHDSI forms a global network allowing for large-scale distributed studies to be performed. A common database management system for instances of the OMOP-CDM is *PostgreSQL* (29). In addition, the following tools are provided for data mapping:

- *WhiteRabbit* is a tool to scan and describe source data.
- *Rabbit in a Hat* supports structural mapping between source data and the OMOP-CDM.
- *USAGI* has been designed to support semantic standardization and terminology mapping.
- *Athena* is as a publicly available web service providing access to the vocabulary used by the OMOP-CDM.

**Figure 1:**
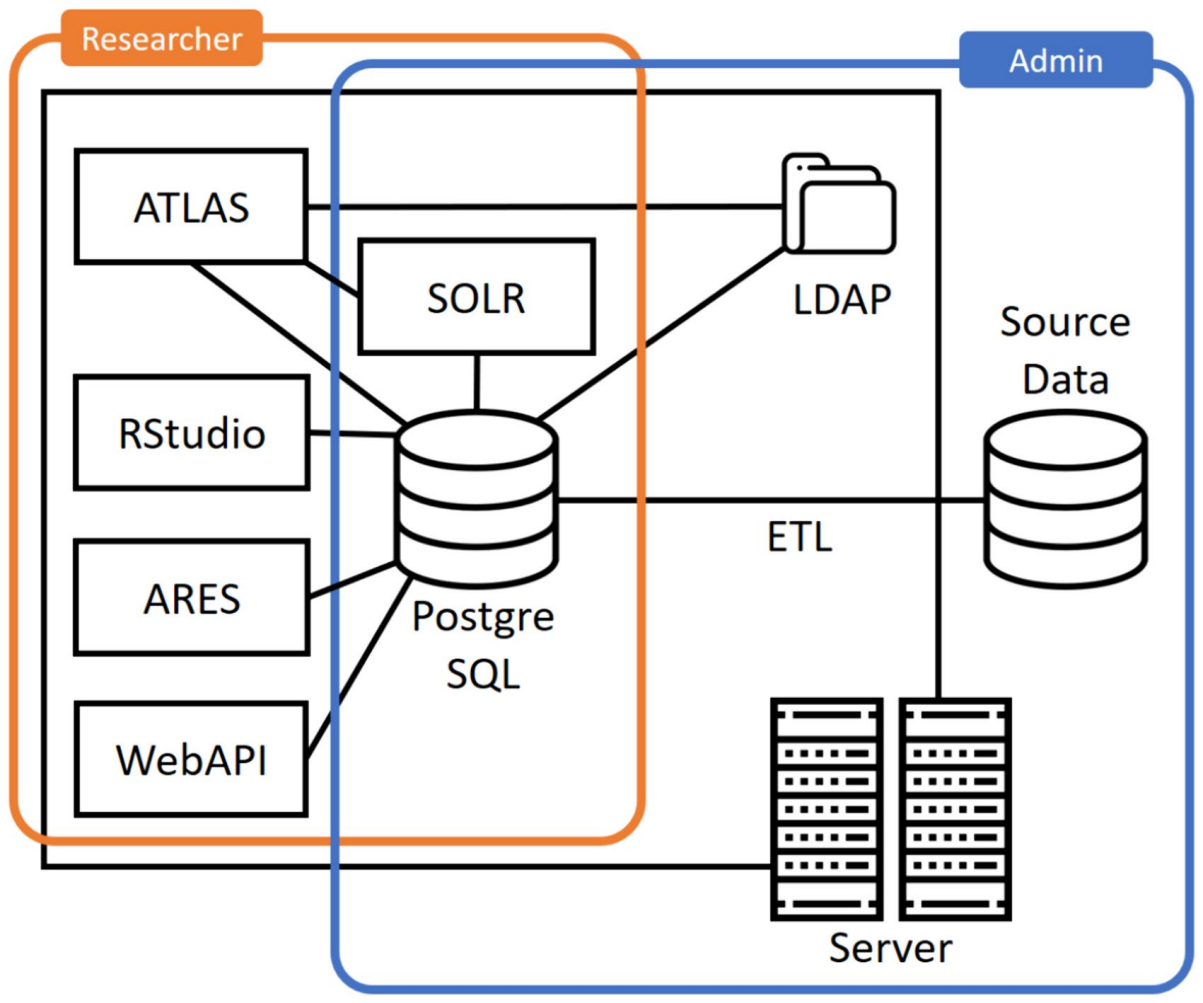
Common architecture of an OHDSI implementation.

We note that OHDSI does not provide a standard tool for extracting, transforming and loading (ETL) data, but focuses on tools for specifying the transformations and mappings needed. A common way of deploying a standard OHDSI stack is the container-based *Broadsea* distribution (30). An overview of a typical set of components in Broadsea is provided in Figure 1.

As can be seen, a common installation contains the following additional infrastructure components:

- A *PostgreSQL* database for storing configuration options and study designs.
- *Apache SOLR* for searching through the vocabulary.
- *OpenLDAP* for authentication and authorization.

Based on this basic infrastructure and the CDM, the Broadsea distribution offers further applications for accessing and analyzing the data:

- *WebAPI* is a RESTful service layer for accessing and analyzing data stored in the OMOP-CDM.
- *ATLAS* is a web-based tool for conducting scientific analyses.
- *ARES* is a system facilitating data exploration, characterization, and quality assessments.
- *RStudio* for analyzing data using the statistical programming language R. Broadsea comes with a range of R-packages, such as Shiny for developing interactive web applications and HADES for analyzing data from the OMOP-CDM.

In summary, researchers can work with data stored in the OMOP-CDM through ATLAS and specific R packages. ATLAS provides graphical access to a variety of OHDSI tools and functions, trading usability off against the flexibility of the analyses that can be performed. In addition, analyses can be performed in R using a set of provided packages and APIs, providing more flexibility in working with the data but requiring programming and data science skills.

### 2.2 Development process

We first identified a set of documents usually required to deploy and operate research systems at German university hospitals. As a basis, these include (1) a description of the processing activities and the technical and organizational measures taken in regards to data protection, (2) an analysis of information security risks and security-related measures taken, (3) a description of processes and responsibilities for maintaining and operating the system. We note that these documents need to be updated regularly following a continuous improvement process.

Next, we related those documents to the systems and processes covered by the common architecture described in the previous section. Data protection aspects were described with a specific focus on systems holding or processing individual-level health data, reflecting requirements by Article 30 GDPR on the content of the description of processing activities. Information security as well as operation of the stack was covered for the complete installation, oriented towards the information security basic protection methodology provided by the German government. Moreover, another document was developed to describe and implement governance processes for use of the data available in the CDM. Finally, we transformed the documents into customizable templates and uploaded them into a version-controlled repository.

## 3 Results

### 3.1 Overview

Table 1 provides an overview of the different document templates developed and provided through a GitHub repository (31).

**Table 1:** Overview of the document templates.

Document title	Short description
Record of processing activities	Description of the data processing activities and protection measures.
Information security concept	Description of information security measures.
Concept of operations	Description of processes and responsibilities when operating the installation.
Authorization concept	Description of groups of user roles and their permissions as well as a description of the process for requesting access to the database.

### 3.2 Record of processing activities

A general description of the software architecture, data flows and processing activities as well as protection measures taken forms the basis of most compliance framework for medical research systems. Thus, as a first component, we developed a template for a Record of Processing Activities (ROPA) for OHDSI installations. As outlined above, ROPAs or related documents are required in most jurisdictions. In this work, we base the content on the requirements outlined in Article 30 of the GDPR and provide information about the personal data processed, the purposes of the processing, retention periods and further relevant details. In the event of legal or data protection audits, the document can be used as a basis to demonstrate compliance and it can also serve as a communication measure for coordinating OHDSI-related activities with an institution’s Data Protection Officer.

### 3.3 Information security concept

While data protection and the ROPA template emphasizes the handling of personal data in a way that respects the rights and expectations of the data subjects, information security focuses on protecting data from unauthorized access and further threats more relevant to the organization itself than to the data subjects. The well-known ISO/IEC 27000 standard emphasizes confidentiality, integrity, and availability, but also adds further aspects, such as authenticity, accountability, non-repudiation, and reliability (32).

To cover these aspects, we provide a template for describing information security-related properties of OHDSI installations. The template is pragmatic and designed to complement existing information security guidelines at the institution operating the installation. It contains a risk analysis of basic processes carried out with OHDSI installations, such as data transformation, loading, and usage, and systematically describes relevant information security measures. As an example, we use modules from the “Basic Protection” methodology of the Federal Office for Information Security in Germany. While there are some differences to the ISO 27000 set of standards, the “Basic Protection” methodology provides a solid foundation of security controls for achieving ISO 27001 compliance. An organization that already applies ISO 27000 can, for example, benefit from our documents through the included risk assessments and lists of relevant security controls that can inform local information security management processes. The document can also support coordination with an institutions Chief Information Security Officer (CISO).

### 3.4 Concept of operations

In addition to a sound and secure setup of an OHDSI node, also the operation of the platform needs to be conceptualized and described. Relevant processes also include the continuous improvement process for data protection and information security-related aspects already described above. In addition, the installed components and their configurations need to be kept up to date, user accounts need to be managed and backups need to be performed. The template for an operational concept includes suggestions for those processes, tailored towards the OHDSI components.

### 3.5 Authorization concept

How access requests by researchers to the OHDSI tools are handled and what governance rules are implemented is an important aspect of compliance. Consequently, we also developed a template for a guideline on how this is implemented. The template describing the access request process describes the duties of administrative personnel responsible for overseeing user access and processes for regular review and removal of outdated permissions. Additionally, it describes the steps researchers must follow to obtain access for conducting studies, including obtaining necessary approvals. In addition to researchers accessing the OHDSI tools, there are further types of personnel involved that need to access the installation for operational purposes. As this is a critical aspect, the proposed template describes all relevant roles, their responsibilities, and access permissions. The template outlines processes for nominating administrators, setting up user access and revoking them upon project completion or staff changes. Moreover, password guidelines and rules for timeouts of sessions are included.

**Figure 2:**
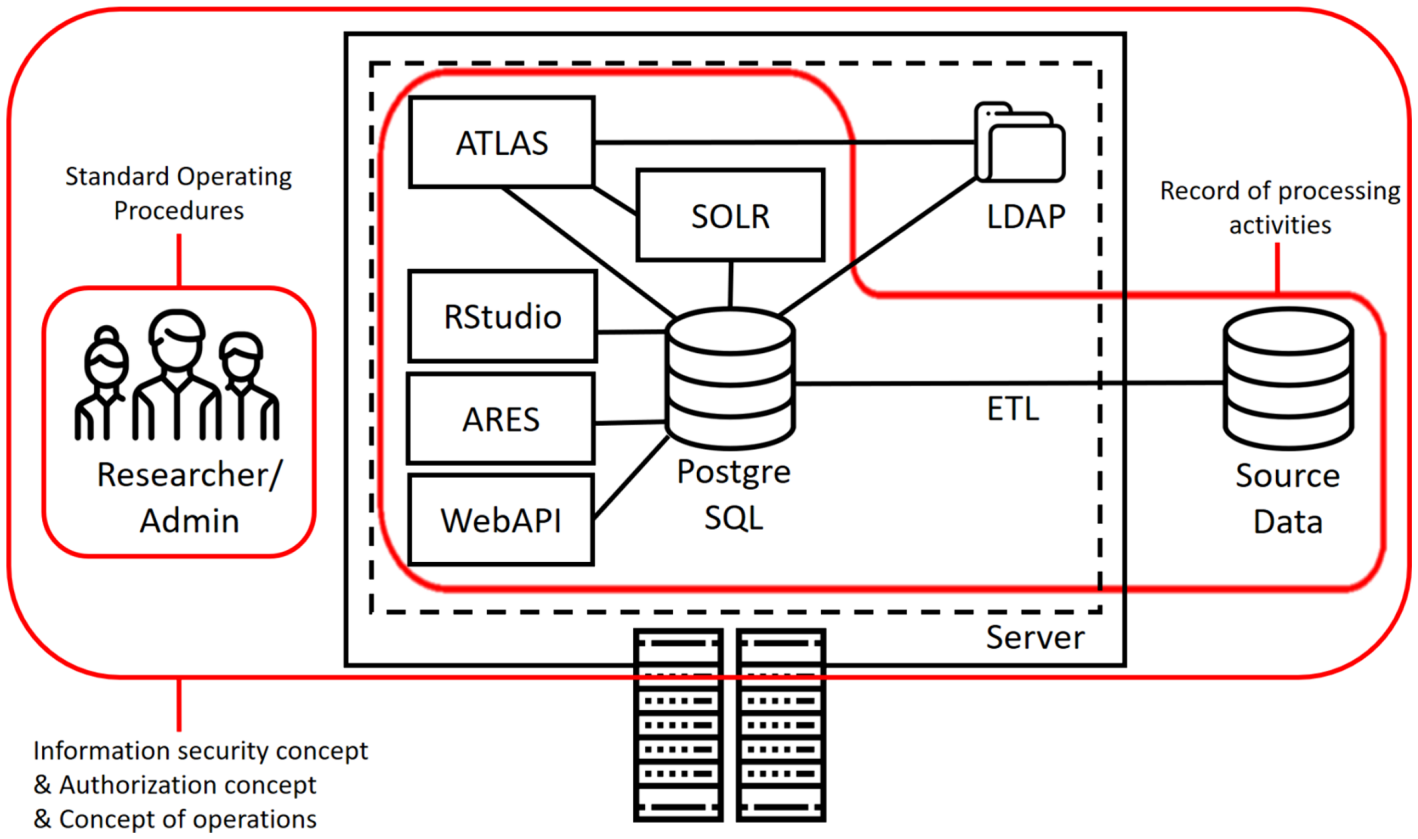
Role of the different documents in a common OHDSI deployment.

Figure 2 illustrates how the developed document templates cover different components and aspects of a common OHDSI installation. As can be seen, the ROPA focuses on the general setup that processes personal data, while the information security concept and related templates cover all components. Access management focuses specifically on humans involved in the maintenance and use of an installation.

### 3.6 Customization and document management

We have developed the templates as Markdown files and provide them in the form of a Git repository. Markdown is a lightweight markup language, designed to be easy to write and read, with the ability to present the document content in many different forms. For example, the documents provided can be compiled into PDF files using open-source tools, such as Pandoc. If visual editing is needed, tools like Pandoc can also be used to convert the markdown files into formats suited for word processors, such as the Open Document Format. We recommend to use the templates in their Markdown version, however, as this naturally enables keeping track of changes in versioned repositories, such as Git.

## 4 Discussion

### 4.1 Principal results

We presented a set of templates for setting up and maintaining OHDSI installations in compliance with data protection and information security requirements, also covering data governance aspects. The document templates are public available under a permissive license. The templates are meant to provide a starting point and need to be filled out accordingly and potentially extended or modified to comply with local policies or legal requirements. We have successfully executed this process at Charité – Universitätsmedizin Berlin.

### 4.2 Comparison with related work

Several institutions or research groups have suggested compliance-oriented document templates for data processing in general or for medical research contexts. Examples include data protection guidelines, see (33) for an example, and templates for institutional review board protocols, see (34) for an example, and information security aspects, see (35) as an example. Quite a lot of the documents are tailored towards specific jurisdictions and published in languages other than English [e.g., (33, 36)]. Our work is different in that it focuses on a typical deployment of a common medical research platform and that its content has been, in large parts, abstracted away from country-specific requirements. Previous work has also focused on compliance for deployments of specific research systems (see the work by Wallace et al. (37) and by Budin-Ljøsne et al. (38) for an example on the DataSHIELD software). To the best of our knowledge, our work is the first to target OHDSI deployments. Governance models have also been studied in the literature. For example, Holmes et al. have presented an overview on governance models for federated research (39). The authors propose a framework with which governance models can be assessed and compared considering different aspects. Pavlenko et al. have focused on data governance for health data warehouses (40).

On a more general level, ethical and legal challenges in data-driven biomedical research have also been studied extensively. For instance, Wang et al. discussed several privacy-enhancing technologies and argue that accountability and informed consent are among the most relevant ethical challenges (41). Arellano et al. conduct a review on privacy regulations, patient perspectives as well as consent practices and their interaction with technology (42). They cover questions, such as under which circumstances consent can be considered ethical. Lamas et al. have argued that ethical and legal frameworks are often not fitting well to common scenarios in the secondary use of health data and the development of health data warehouses (43).

Kalkman et al. have studied the sharing practices for compliance-related documentation (44). The authors found that documents like the ones presented in this work is not common.

The OHDSI software stack addressed in the work described in this paper, is expected to play an important role in the upcoming EHDS and is promoted by a range of institutions. For example, the DARWIN initiative - an infrastructure built by the European Medicines Agency (EMA) to enable the secondary use of real-world data - is based on the OMOP-CDM and can be considered one of the first functional parts of the EHDS (45). The Joint Action Towards the European Health Data Space (TEHDAS) is another project with significant contributions to the shaping of the EHDS. Recently, also Health Level Seven (HL7) International and OHDSI have started a collaboration to work on a joint common data model for sharing information for healthcare and research (46).

### 4.3 Limitations and future work

One limitation of our work is that it has been designed with European and German requirements in mind, although we aimed at generalizing and abstracting away specifics. We note, however, that there are many similarities between relevant laws and regulations in different parts of the world (*cf.* similarities between the California Consumer Privacy Act or the EU-US Data Privacy Framework and the GDPR). We stress again that our templates must hence be regarded as a starting point and might need adaptions. In future work, we hope to be able to extend and adjust our templates based on feedback from their application in different contexts and jurisdictions.

Another limitation of our work is that we currently did not explicitly include a document template for a Data Protection Impact Assessment (DPIA). Under the GDPR a DPIA is necessary for processing activities resulting in a high risk for the privacy of the data subjects. If an institution decides that this is needed for an OHDSI installation, tools, such as the one presented in (47), can be used and information from the documents provided through our work can be reused.

One interested area for future work is to more thoroughly study the compliance of data sharing processes within the OHDSI network. For example, it is not trivial to decide when aggregated statistics can be considered to be anonymous data. The OHDSI collective could be supported by a guideline providing legal and technical assessments of commonly used methods.

## 5 Summary and conclusion

In this paper, we introduced a set of document templates designed to facilitate the implementation and operation of an OHDSI software stack for generating real-world evidence in compliance with data protection and information security requirements. These templates, tailored for typical OHDSI deployments, include crucial documents, such as a Record of Processing Activities, an Information Security Concept, and an Operational Concept. Our work addresses a significant gap by providing a framework adaptable to different institutional and legal requirements, thereby simplifying compliance processes for OHDSI deployments. Despite being primarily oriented towards European and German regulations, our templates can serve as an adaptable starting point for organizations worldwide. Future efforts will focus on refining these templates based on feedback received and extending their scope to further compliance aspects.

## Data availability statement

The datasets presented in this study can be found in online repositories. The names of the repository/repositories and accession number(s) can be found below: The templates created can be found in the associated GitHub repository: https://github.com/BIH-MI/ohdsi-compliance.

## Author contributions

FW: Conceptualization, Resources, Writing – original draft, Writing – review & editing. HA: Resources, Writing – original draft, Writing – review & editing. FP: Conceptualization, Resources, Writing – original draft, Writing – review & editing.
